# Effects of Storage Temperature and pH on the Antifungal Effects of Commercial Oral Moisturizers against *Candida albicans* and *Candida glabrata*

**DOI:** 10.3390/medicina56100525

**Published:** 2020-10-07

**Authors:** Mamoru Murakami, Kae Harada, Yasuhiro Nishi, Takaharu Shimizu, Sara Motoyama, Masahiro Nishimura

**Affiliations:** 1 Denture Prosthodontics Restoration, Advanced Dentistry Center, Kagoshima University Hospital, 8-35-1 Sakuragaoka, Kagoshima 890-8544, Japan; 2 Departments of Oral and Maxillofacial Prosthodontics, Field of Oral and Maxillofacial Rehabilitation, Advanced Therapeutic Course, Kagoshima University Graduate School of Medical and Dental Sciences, 8-35-1 Sakuragaoka, Kagoshima 890-8544, Japan; hkae@dent.kagoshima-u.ac.jp (K.H.); shar@dent.kagoshima-u.ac.jp (Y.N.); dentshimizu@me.com (T.S.); mnishi@dent.kagoshima-u.ac.jp (M.N.); 3 Section of Dental Hygiene, Division of Clinical Technology, Kagoshima University Hospital, 8-35-1 Sakuragaoka, Kagoshima 890-8544, Japan; k9604974@dent.kagoshima-u.ac.jp

**Keywords:** oral moisturizer, *C. albicans*, *C. glabrata*, pH, xerostomia

## Abstract

*Background and objectives*: Oral moisturizers have been used to treat dry mouth. This study aimed to investigate the effects of storage temperature and pH on the antifungal effects of oral moisturizers against *Candida albicans* and *Candida glabrata*. *Materials and Methods*: Thirty-one oral moisturizers and amphotericin B (AMPH-B) were stored at 25 and 37 °C for 1 week. Subsequently, they were added to cylindrical holes in 50% trypticase soy agar plates inoculated with *C. albicans* and *C. glabrata* (10^7^ cells/ml). The antifungal effects were evaluated based on the sizes of the growth-inhibitory zones formed. Two-way analysis of variance was used to determine the effects of storage temperature and pH on the growth-inhibitory zones. *Results*: Significant differences in the effects of storage temperature and pH of the moisturizers were observed against *C. albicans* and *C. glabrata*. The growth-inhibitory zones of samples stored at 37 °C and with neutral pH were significantly larger than those stored at 25 °C and with acidic pH, respectively. The sizes of the zones formed by most of the oral moisturizers were larger than those formed by AMPH-B (concentration, 0.63 µg/ml). *Conclusion*: The antifungal effects of oral moisturizers against *C. albicans* and *C. glabrata* were affected by their storage temperature and pH.

## 1. Introduction

The number and detection rate of *Candida* are increased in the oral cavity and on the surface of dentures in patients with dry mouth due to decreased salivary secretion [1,2,3,4]. *Candida* co-aggregates with highly pathogenic bacteria and creates a bacterial reservoir [5,6] leading to oral candidiasis and denture stomatitis [3,5,6]. Oral candidiasis is an opportunistic infection caused by *Candida albicans* in approximately 80% of cases [7,8]. On the other hand, *Candida glabrata* has been detected in immunocompromised patients with head and neck cancer and HIV, and those with denture stomatitis [9,10,11]. However, an increase in the number of patients with *C. glabrata* infection and oral candidiasis has been reported recently [8,9,12,13,14]. Oral candidiasis caused by *C. glabrata* is intractable because *C. glabrata* has intrinsically low susceptibility and is able to easily become resistant to azole antifungal agents, thus making it difficult to treat [13,15,16].

Although antifungal agents are used to treat oral candidiasis and denture stomatitis, long-term use of antimicrobial agents with a broad antifungal spectrum should be avoided due to the emergence of drug-resistant strains [17]. Dry mouth is a chronic disease; consequently, it is important to select a drug that can be used continuously for a long time to treat the oral candidiasis that accompanies this condition [18]. Therefore, oral moisturizers with antifungal effects may be useful for the symptomatic treatment of dry mouth. A few studies on the antifungal effect of oral moisturizers have been conducted so far, and the results are inconsistent [19,20,21,22].

The optimum temperature and pH for the growth of *Candida* are lower than those required for other bacteria [23,24]. The heating of an antifungal drug (fluconazole) has been shown to significantly increase its ability to inhibit biofilm formation by *C. albicans* [25,26]. Generally, the pH of a substance changes depending on the temperature, but the pH of commercially available oral moisturizers varies greatly depending on the type of product [27]. Therefore, the storage temperature and pH of an oral moisturizer might influence its antifungal effect.

The purpose of this study was to examine the effects of storage temperature and pH on the antifungal effects of oral moisturizers against *C. albicans* and *C. glabrata*.

## 2. Materials and Methods

### 2.1. Samples

Thirty-one commercially available oral moisturizers (11 liquid- or spray-type and 20 gel-type) along with amphotericin B (AMPH-B, Sigma-Aldrich Japan, Tokyo, Japan), dimethyl sulfoxide (DMSO), and distilled water were used in this study. AMPH-B and distilled water were used as positive and negative controls, respectively. The final concentration of AMPH-B with dimethyl sulfoxide was 1.25–0.04 µg/ml (DMSO%; 99.50–99.98%). Table 1 (liquid or spray) and Table 2 (gel) present the details of the moisturizers used in this study. Unopened oral moisturizers were heated in a direct heat CO_2_ incubator (#310, Thermo Fisher Scientific KK, Tokyo, Japan) at 37 °C and a cool incubator (KMH-050, AS ONE Ltd., Osaka, Japan) at 25 °C, starting from 1 week before the experiment.

### 2.2. Evaluation of Antifungal Effects

*C. albicans* (JCM1537, Riken BRC, Tsukuba, Japan) and *C. glabrata* (JCM3699, Riken BRC, Tsukuba, Japan) were cultured in Tryptic Soy Broth (TSB: Becton, Dickinson and Company, France. 0.8% agar: CAG5858, WAKO, Japan) liquid medium (5 ml) at 37 °C for 24 h, respectively. *C. albicans* and *C. glabrata* at concentrations of 10^7^ cells/ml (100 μl) were mixed with 5 ml of TSB agar media and inoculated on 50% TSA plates (TSB: 15g/l, 2% agar) [18]. After the hardening of the media, 20-µl samples were placed within cylindrical holes (diameter, 5 mm; depth, 5 mm) that were prepared in the TSA plates [18]. The diameters of the growth-inhibitory zones were measured after 24 h. The growth-inhibitory zones for each sample were obtained from five plates and the average value was measured.

### 2.3. pH Measurement

The pH values of the oral moisturizers with antifungal effect were measured with a glass electrode (9681S-10D, HORIBA, Kyoto, Japan) connected to a portable pH meter (D-72, HORIBA, Kyoto, Japan) at 25 and 37 °C. The pH meter had a measurement range of pH 0 to 14, resolution of pH 0.01, and measurement accuracy of pH ± 0.01. The pH meter was calibrated using three types of calibration standard solutions (pH 4.01, pH 7.00, and pH 10.01) before the actual pH measurements.

The pH electrode was inserted into an 8-ml tube (Sarstedt K. K., Tokyo, Japan) containing 4 ml of the sample and held for 2 min. The same sample was measured five times, and the average value was calculated. The pH values obtained were classified as acidic if <6.5, and neutral if ≥6.5 and <7.5.

### 2.4. Statistical Analysis

A Chi-square test was used to compare the categorical variables. A *t*-test was used to compare the variables between the *Candida* species. The effects of pH type (neutral or acid) and storage temperature (25 or 37 °C) on the sizes of the growth-inhibitory zones were evaluated using the analysis of variance (ANOVA) test. The sizes of the growth-inhibitory zones were analyzed using Tukey’s multiple comparisons test. All statistical analyses were performed using IBM SPSS Statistics 26 (Japan IBM, Tokyo, Japan), and the significance level was set a *p* < 0.05.

## 3. Results

### 3.1. Measurement Results of Each Moisturizer at 25 and 37 °C

Table 3 shows the size of the growth-inhibitory zone (mm) against *C. albicans*, pH value, and pH type of each sample at 25 and 37 °C. A total of eight oral moisturizers with antifungal effects against *C. albicans*, including two gel types and six liquid types, were observed. The average sizes of the growth-inhibitory zones of the liquid- and gel-type moisturizers were 7.66 and 8.72 mm at 25 °C, respectively, and 10.22 and 11.61 mm at 37 °C, respectively. The average pH value of the samples with growth-inhibitory zones against *C. albicans* at 25 °C was 6.78 for the liquid-type moisturizers and 6.38 for the gel-type moisturizers; the corresponding values at 37 °C were 6.45 and 6.41 for the liquid- and gel-type moisturizers, respectively. The proportion of moisturizers with an acidic pH was 56.3% (9/16).

Table 4 shows the size of the growth-inhibitory zone (mm) against *C. glabrata*, pH value, and pH type of each sample at 25 and 37 °C. A total of 12 oral moisturizers with antifungal effects against *C. glabrata*, including three gel types and nine liquid types, were observed. The eight moisturizers demonstrated antifungal effects against both *C. albicans* and *C. glabrata*. The average sizes of the growth-inhibitory zones for the liquid- and gel-type moisturizers were 12.24 and 11.68 mm at 25 °C, respectively, and 15.50 and 15.06 mm at 37 °C, respectively. The average pH value of the samples with growth-inhibitory zones against *C. glabrata* at 25 °C was 6.70 for the liquid-type moisturizers and 5.93 for the gel-type moisturizers; the corresponding values at 37 °C were 6.45 for the liquid-type moisturizers and 5.96 for the gel-type moisturizers. The proportion of moisturizers with an acidic pH was 66.7% (16/24).

### 3.2. Differences in Candida Species and Antifungal Effects of Oral Moisturizers

The upper part of Table 5 shows the number of growth-inhibitory zones against *C. albicans* and *C. glabrata* for each type of oral moisturizer. The results of the *χ*^2^ test indicated a significant difference in the number of growth-inhibitory zones and the *Candida* species, regardless of the type of oral moisturizer used. The lower part of Table 5 shows the mean size of the growth-inhibitory zone and mean pH value against *C. albicans* and *C. glabrata*. The size of the growth-inhibitory zone against *C. albicans* was significantly smaller than that against *C. glabrata*, and the pH value of the moisturizers that demonstrated an antifungal effect against *C. albicans* was significantly higher than that against *C. glabrata* (*t*-tests).

### 3.3. The Effects of pH Type and Storage Temperature on the Sizes of the Growth-Inhibitory Zones

Table 6 show the results of two-way analysis of variance (ANOVA) for the effects of the storage temperature and pH type of the oral moisturizers on the size of the growth-inhibitory zone of the oral moisturizer against *C. albicans* and *C. glabrata*, respectively. The results indicate storage temperature (A) and pH type (B) had significant effects on the sizes of the growth-inhibitory zones (*p* < 0.05) against both *C. albicans* and *C. glabrata*; however, the interaction between them (A × B) was not significant. The size of the growth-inhibitory zone against *C. albicans* was significantly larger at a storage temperature of 37 °C (11.26 mm) than that at 25 °C (8.64 mm) and was significantly larger in the neutral pH products (10.58 mm) than in the acidic pH products (9.33 mm). For *C. glabrata*, the size of the growth-inhibitory zone at 37 °C was significantly larger (15.30 mm) than that at 25 °C (12.03 mm); likewise, it was significantly larger in the neutral pH products (14.18 mm) than in the acidic pH products (13.15 mm).

### 3.4. Comparison of the Growth-Inhibitory Zones between Oral Moisturizers and AMPH-B

Comparisons of the sizes of the growth-inhibitory zones among the various concentrations of AMPH-B and oral moisturizers used against *C. albicans* and *C. glabrata*, respectively, are shown in Table 7 and Table 8. The value in the box represents the p-value of the Tukey test, and in the horizontal direction, * represents that the size of growth-inhibitory zone of AMPH-B was larger than that of the oral moisturizer, and # represents the opposite. The bold and italic values indicate that the size of the growth-inhibitory zones of the oral moisturizer is equal or greater than that of AMPH-B.

In Table 7, the gel-type moisturizer G presented with the largest the growth-inhibitory zone at 25 °C, larger than that observed with AMPH-B (concentration, 0.63 µg/ml); however, the sizes of the zones in the cases of most of the other moisturizers were the equal to or less than that observed with AMPH-B (concentration, 0.08 µg/ml). At 37 °C, the sizes of the growth-inhibitory zones were increased with all the moisturizers, and many of them were larger than those observed with AMPH-B at a concentration of 0.63 µg/ml.

As seen in Table 8, the gel-type moisturizer G presented with the largest growth-inhibitory zone against *C. glabrata* at 25 °C, larger than that observed with AMPH-B (concentration, 0.63 µg/ml); however, the sizes of the zones with the other moisturizers were equivalent to that observed with AMPH-B (concentrations, 0.31 µg/ml to 0.04 µg/ml). At 37 °C, the sizes of the growth-inhibitory zones of all the moisturizers were increased to those equivalent to or larger than that observed with AMPH-B (concentration, 0.63 µg/ml).

## 4. Discussion

It is important to avoid the risk of systemic infections, which can lead to poor quality of life in patients with dry mouth, due to the increased levels of *Candida* in the oral cavity and mucosal surfaces of dentures [1,2,3,4]. Although antifungal agents are prescribed for the treatment of oral candidiasis, azole antifungal agents are contraindicated in patients receiving anticoagulant therapy; moreover, long-term use of antifungal agents should be avoided due to the emergence of drug-resistant strains [17]. However, considering that oral moisturizers are routinely used for patients with dry mouth, the use of a moisturizer with antifungal effects might prove effective in reducing the risk of *Candida* infection. Therefore, this study comprehensively investigated the antifungal effects of commercially available oral moisturizers against *C. albicans* and *C. glabrata* and examined the effects of storage temperature and pH on the antifungal effects of these moisturizers.

It is not clear that the agar diffusion method, employed to test antifungal proficiency, accurately models in vivo activity. Since the agar diffusion method could be affected by differences in viscosity, differences in viscosity of oral moisturizer may affect differences in the zones of inhibition [28,29,30]. Additionally, a moisturizer stored at 25 °C should quickly ascend to a much higher temperature in the mouth. The concentration of AMPH-B used in this study was determined with reference to previous in vitro studies [18]. However, the in vitro results can be extrapolated in in vivo conditions with certain limitations.

Physical properties, such as viscosity, transpiration rate, and adhesive strength of oral moisturizers used for the symptomatic treatment of patients with dry mouth [1,2,18], have been studied previously [27,28,29]. However, reports on the antibacterial properties of these moisturizers are limited [18,19,20,21,22]. The efficacy of oral moisturizing jelly (OMJ) and a topical commercial gel (GC dry mouth gel) on *Candida* colonization and saliva properties was evaluated by a randomized controlled trial [22]. The results show that both OMJ and GC saliva gels could improve saliva pH and decrease the number of *Candida* species [22]. The current study was the first to comprehensively investigate the antifungal properties of commercially available oral moisturizers against *C. albicans* and *C. glabrata*.

In the present study, the antifungal effects observed against *C. glabrata* were higher than those observed against *C. albicans*, and the sizes of the growth-inhibitory zones against *C. glabrata* were significantly larger than those against *C. albicans*. Oral candidiasis is a fungal disease mainly caused by *C. albicans* [7,8]. However, an increase in the involvement of *C. glabrata* has been reported over the past few years [8,9,12,13,14]. The adhesive strength of *C. glabrata* to acrylic resin is twice that of *C. albicans* [7]; therefore, it is more frequently isolated from the mucosal surfaces of dentures in elderly patients [31]. *C. glabrata* cannot invade the epithelium by itself because it is a non-dimorphic yeast, but co-infection with *C. albicans* promotes invasion into the epithelium [9,32]. The proportion of *C. glabrata* resistant to azole antifungal agents is increasing [13]. In addition, there are reports that infection with *C. glabrata* alone or in combination with *C. albicans* is more resistant to azole antifungal agents than that with *C. albicans* alone [10,11]. In this study, the antifungal effects on *C. albicans* and *C. glabrata* were individually investigated. However, additional studies are required to examine the co-cultures of these species in the future.

The labels on oral moisturizers contain instructions about the storage of the product away from high temperatures and direct sunlight, but clear instructions about the appropriate storage temperature are not specified generally. In this study, the storage temperature of the oral moisturizers was set based on room temperature and body temperature. The heating of fluconazole at 39 °C was shown to significantly increase its ability to inhibit biofilm formation by *C. albicans* when compared to that at 37 °C [26]. Although it was not the same as the heating temperature of fluconazole, the sizes of the growth-inhibitory zones formed by moisturizers stored at 37 °C were significantly larger than those formed by moisturizers that were stored at 25 °C in the current study. This may be attributed to the fact that the optimum temperature for *Candida* growth is 25 to 30 °C, which is lower than that for other bacteria [23,24]. This difference may have affected the activity of the antifungal ingredients in the oral moisturizers.

In this study, the oral moisturizers with antifungal effect contained an egg yolk antibody against *Candida,* in addition to hinokitiol, whey protein, and lactoferrin [18,33]. The differences in the sizes of the growth-inhibitory zones between the different concentrations of AMPH-B and the various oral moisturizers were attributed to the different mechanisms of actions of the antifungal ingredients contained in the moisturizers. Moisturizer G, which had the highest antifungal activity, contained hinokitiol. Hinokitiol is obtained from Hiba (a type of cypress) and exerts high antibacterial activity by inhibiting the tricarboxylic acid cycle of the bacteria [18]. On the other hand, egg yolk antibody, which is included in gel-type moisturizers “H” and “N”, binds to the adhesion factor and the toxin produced by *Candida*, thereby inhibiting its adhesion to mucosal epithelial cells and inactivating the toxin [33]. Whey protein, which is present in the liquid-type moisturizer “c” and gel-type moisturizer “J”, should be used with caution in patients with a history of milk allergy [18].

The antifungal effects of oral moisturizers at temperatures other than those described in this study remain unknown because they have not been evaluated so far. The results of a study in which the antifungal properties of fluconazole were increased by heating it to 39 °C [26] suggest that it is necessary to investigate the antifungal properties of oral moisturizers stored at temperatures above 37 °C. However, if the oral moisturizer is stored at a high temperature for a long time, a deterioration in its quality may be expected. In a previous study, the antimicrobial activity of an oral moisturizer stored in an incubator at 37 °C for 8 h was significantly lower than that observed immediately after opening the package [18]. This result suggests that when heating the oral moisturizer, it is necessary to devise a heating method such as heating immediately before use.

The pH of saliva is reduced in patients with dry mouth [1,2,33]. Consequently, the pH is optimum for the secretion of aspartyl proteinase produced by *Candida,* which leads to an increase in its pathogenicity [34]. The detection of significantly larger growth-inhibitory zones in moisturizers with neutral pH compared to those with acidic pH in the current study corresponded with the fact that the optimum pH of *Candida* is 5.6, which is lower than that of other bacteria [21,24,35]. The critical pH values at which enamel dissolves range from 5.5 to 6.5, depending on the phosphate and calcium concentrations in saliva [36]. In the present study, a pH lower than 6.5 was defined as acidic based on the critical pH value when the concentrations of phosphate and calcium were high. According to the results of this study, the pH values of oral moisturizers with antifungal effects ranged from 3.46 to 7.29, but about 60% of them were more acidic than the critical pH value. These findings are largely consistent with the results of a survey of seven commercially available oral moisturizers [27]. Taken together, these results suggest that caution should be exercised when using oral moisturizers in dentulous patients with dry mouth because moisturizers with an acidic pH have low antifungal activity and the potential to erode teeth [27].

In this study, the number of oral moisturizers investigated greatly differed from those in past reports, which may be attributed to the product life cycle of the moisturizers. The product life cycle of oral moisturizers in Japan is short, and only 12 products that were used in a previous survey were used in the current study [18]. Oral moisturizers are used as self-care products for patients who complain of dry mouth and are purchased by individuals based on their preference. Therefore, it is necessary for medical staff to fully understand the symptoms of the patient and the characteristics of the oral moisturizer used, particularly regarding the selection and storage of the oral moisturizer. Furthermore, it is important to constantly update the information and provide appropriate advice on the selection and usage of these moisturizers.

## 5. Conclusions

Within the limitations of this in vitro study, our findings suggested that the antifungal effects of oral moisturizers against *C. albicans* and *C. glabrata* were affected by their storage temperature and pH.

## Figures and Tables

**Table 1:** Details of the liquid- and spray-type moisturizers used in this study.

Materials	Code	Manufacturer	Main/Active Ingredients
Stoppers for	a	Sun Dental Co., Ltd.	Water, Glycerin, Xylitol, Lysozyme, Lactoferrin
Oral Wet Spray	b	Yoshida Co., Ltd.	Water, Xylitol, Sodium benzoate, Hyaluronate sodium
ConCool Mouth Rinse	c	Weltec Co., Ltd.	Water, PG, Sorbitol, Xylitol, Lactoferrin, Whey protein
Pepti Sal Mouth Wash	d	T&K Co., Ltd.	Water, PG, Xylitol, Polyglycitol, Nisin, Lactoferrin
Wet Keeping Mist	e	Oral Care. Inc.	Glycerin, Betaine, Xylitol, Methylparaben, Sodium citrate
Biotene Mouth Wash	f	GSK Co., Ltd.	Water, Glycerin, Xylitol, Sorbitol, Propylene glycol
Clean & Moisture Spray	g	Trife Inc.	Water, Glycerin, Lactococcus culture extract, Ume extract
Lion Oral Spray	h	Lion Corporation	Glycerin, PG, Polyglutamic acid, Xylitol, l-menthol
Care Heart Spray	i	Tamagawa Eizai Co., Ltd.	Water, Glycerin, Xylitol, Sodium citrate
SMILE HONEY Gel Spray	j	Nippon Zettoc Co., Ltd.	Water, Glycerin, Propanediol, Hyaluronate sodium, Xylitol
Sunstar Gel Spray	k	SUNSTAR Co., Ltd.	Water, Glycerin, Glycosyl trehalose, BG
Otsuka distilled water	w	Otsuka Pharmaceutical	Distilled water
Amphotericin B	AMPH-B	Wako	Polyene antimycotic
Dimethyl sulfoxide	DMSO	Sigma-Aldrich	(CH_3_)_2_SO, CAS No. 67- 68- 5

**Table 2:** Details of the gel-type moisturizers used in this study.

Materials	Code	Manufacturer	Main/Active Ingredients
Wet aid	A	Dent care. Inc.	Water, Maltitol, Trehalose, BG, Hyaluronate sodium
Oral Moisturizer Ai Gel	B	Ryoka Dental. Inc.	Water, Glycerin, Carboxymethyl cellulose, Xylitol
Oral Aquagel	C	GC Co., Ltd.	Diglycerine, Water, Carboxymethyl cellulose
Denture Gel	D	Kamemizu Chem. Ind.	Maltitol, Water, Glycerin, Propylene glycol
Ulora Gel	E	BEE BRAND MEDICO DENTAL.CO., LTD.	Water, Glycerin, Xylitol, Sorbitol, Propylene glycol
Wet Keeping	F	Oral Care. Inc.	Water, Glycerin, Betaine, Xylitol, Hydroxyethyl cellulose
Refre-care	G	EN Otsuka Pharmaceutical	Water, Glycerin, Hyaluronate sodium, Hinokitiol, Xylitol
SMILE HONEY Oral Clean Gel	H	Nippon Zettoc Co., Ltd.	Water, Sorbitol, Glycerin, Xanthan gum, Dried egg yolk
SMILE HONEY Honey Gel	I	Nippon Zettoc Co., Ltd.	Honey, Xylitol, Sucralose, Lemon juice, Tea extract
ConCool Mouth Gel	J	Weltec Co., Ltd.	Water, Maltitol, Sorbitol, Glycerin, Xylitol, Whey protein
Oral Balance Gel	K	GSK Co., Ltd.	Glycerin, Water, Sorbitol, Xylitol, Hydroxyethyl cellulose
Mouth Moist	L	Osaki Medical Co., Ltd.	Water, Glycerin, Cellulose gum, Hyaluronate sodium
Pepti Sal Gentle Mouth Gel	M	T&K Co., Ltd.	Glycerin, Polyethylene glycol, Xylitol, Water, Lactoferrin
Terumo Oral Gel	N	Nippon Zettoc Co., Ltd.	Water, Sorbitol, Glycerin, Xylitol, Dried egg yolk, Maltitol
Mouth Pure Oral care gel	O	Kawamoto Corporation	Water, Glycerin, Betaine, Hyaluronate sodium, Sodium polyacrylate
Butler Moisturizing Transparent Gel	P	SUNSTAR Co., Ltd.	Water, Glycerin, Sorbitol, Hydrogenated starch hydrolysate, Propylene glycol
Viva-Jellwet	Q	Nippon Zettoc Co., Ltd.	Water, Glycerin, Sodium alginate, Hydroxyethyl cellulose, Cetylpyridinium chloride
Clean & Moisture Gel	R	Trife Inc.	Glycerin, Water, Lactococcus culture extract, Ume extract
OKUCHI WO ARAU GEL	S	Nippon Shika Yakuhin Co., Ltd.	Water, Glycerin, Hydroxyethyl cellulose, Hyaluronate sodium
Care Heart Gel	T	Tamagawa Eizai Co., Ltd.	Water, Glycerin, Xylitol, Hydroxyethyl cellulose

**Table 3:** Mean and standard deviation of the growth-inhibition zone (mm), pH value, and pH type of the moisturizers against *Candida albicans*.

	25 °C			37 °C				25 °C			37 °C		
Code	GIZ (SD)	pH Value	pH Type	GIZ (SD)	pH Value	pH Type	Code	GIZ (SD)	pH Value	pH Type	GIZ (SD)	pH Value	pH Type
a	0			0			A	0			0		
b	0			0			B	0			0		
c	8.01 (0.11)	7.12 (0.11)	Na	10.45 (0.16)	6.36 (0.01)	Ac	C	8.57 (0.12)	7.29 (0.00)	Na	11.16 (0.11)	7.10 (0.01)	Na
d	0			0			D	0			0		
e	0			0			E	0			0		
f	0			0			F	0			0		
g	0			0			G	11.55 (0.12)	6.64 (0.00)	Na	15.00 (0.07)	6.73 (0.00)	Na
h	0			0			H	8.94 (0.12)	5.62 (0.00)	Ac	11.39 (0.08)	5.69 (0.00)	Ac
i	0			0			I	0			0		
j	7.22 (0.11)	6.44 (0.11)	Ac	9.98 (0.10)	6.53 (0.00)	Na	J	0			0		
k	0			0			K	0			0		
w	0			0			L	0			0		
							M	0			0		
							N	0			0		
							O	7.81 (0.08)	6.40 (0.00)	Ac	10.58 (0.07)	6.47 (0.00)	Ac
							P	0			0		
							Q	7.76 (0.11)	5.86 (0.01)	Ac	10.68 (0.10)	5.93 (0.00)	Ac
							R	0			0		
							S	7.66 (0.08)	6.48 (0.00)	Ac	10.85 (0.03)	6.53 (0.00)	Na
							T	0			0		
Average	7.66 (0.43)	6.78 (0.32)		10.22 (0.26)	6.45 (0.08)			8.72 (1.33)	6.38 (0.53)		11.61 (1.52)	6.41 (0.47)	

GIZ—growth-inhibition zone (mm); SD—standard deviation; pH type (Neutral—Na; Acid—Ac).

**Table 4:** Mean and standard deviation of the growth-inhibition zone (mm), pH value, and pH type of the moisturizers against *Candida glabrata*.

	25 °C			37 °C				25 °C			37 °C		
Code	GIZ (SD)	pH Value	pH Type	GIZ (SD)	pH Value	pH Type	Code	GIZ (SD)	pH Value	pH Type	GIZ (SD)	pH Value	pH Type
a	0			0			A	0			0		
b	0			0			B	0			0		
c	12.40 (0.07)	7.12 (0.11)	Na	15.77 (0.05)	6.36 (0.01)	Ac	C	10.32 (0.04)	7.29 (0.00)	Na	14.57 (0.10)	7.10 (0.01)	Na
d	0			0			D	0			0		
e	0			0			E	10.50 (0.07)	3.58 (0.01)	Ac	14.57 (0.07)	3.46 (0.00)	Ac
f	0			0			F	0			0		
g	0			0			G	15.61 (0.14)	6.64 (0.00)	Na	18.58 (0.14)	6.73 (0.00)	Na
h	12.36 (0.03)	6.55 (0.01)	Na	15.89 (0.04)	6.45 (0.00)	Ac	H	11.93 (0.07)	5.62 (0.00)	Ac	14.93 (0.07)	5.69 (0.00)	Ac
i	0			0			I	0			0		
j	11.97 (0.06)	6.44 (0.11)	Ac	14.84 (0.03)	6.53 (0.00)	Na	J	0			0		
k	0			0			K	0			0		
w	0			0			L	11.41 (0.04)	5.97 (0.00)	Ac	14.41 (0.04)	6.06 (0.00)	Ac
							M	0			0		
							N	10.84 (0.06)	5.54 (0.01)	Ac	14.33 (0.04)	5.69 (0.00)	Ac
							O	11.87 (0.03)	6.40 (0.00)	Ac	14.52 (0.15)	6.47 (0.00)	Ac
							P	0			0		
							Q	11.86 (0.04)	5.86 (0.01)	Ac	14.86 (0.04)	5.93 (0.00)	Ac
							R	0			0		
							S	10.77 (0.16)	6.48 (0.00)	Ac	14.76 (0.16)	6.53 (0.00)	Na
							T	0			0		
Average	12.24 (0.20)	6.70 (0.29)		15.50 (0.46)	6.45 (0.07)			11.68 (1.49)	5.93 (0.97)		15.06 (1.25)	5.96 (0.98)	

GIZ—growth-inhibition zone (mm); SD—standard deviation; pH type (Neutral—Na; Acid—Ac).

**Table 5:** Number of growth-inhibition zones against *C. albicans* and *C. glabrata* with each oral moisturizer (upper row) and sizes of growth-inhibition zones and pH values in *C. albicans* and *C. glabrata* (lower row).

Type of Moisturizer		*C. Glabrata* (+)	*C. Glabrata* (−)	Total		*p*-Value
Liquid	*C. albicans* (+)	2	0	2		
	*C. albicans* (−)	1	8	9		
Subtotal		3	8	11		0.04 *
Gel	*C. albicans* (+)	6	0	6		
	*C. albicans* (−)	3	11	14		
Subtotal		9	11	20		0.00 *
Total	*C. albicans* (+)	8	0	8		
	*C. albicans* (−)	4	19	23		
	Total	12	19	31		0.00 *
* *p* < 0.05 denotes significant difference: Chi-square test						
Type of Fungi	Mean GIZ (SD)	95% CI	*p-*value	Mean pH value (SD)	95% CI	*p*-value
*C. albicans*	9.86 (0.23)	9.41–10.31	0.00 *	6.45 (0.51)	6.33–6.57	0.00 *
*C. glabrata*	13.50 (0.19)	13.13–13.86		6.07 (0.94)	5.89–6.25	

GIZ—growth-inhibition zone (mm); * *p* < 0.05 denotes significant difference: *T*-tests.

**Table 6:** Results of the analysis of variance test for *C. albicans* and *C. glabrata*.

Source	d. f.	Sum of Squares	Mean Square	*f*-Value	*p*-Value
*C. albicans*					
Temperature (A)	1	132.78	132.78	83.36	0.00 *^a^
pH type (B)	1	30.12	30.12	18.91	0.00 *^b^
A × B	1	1.46	1.46	0.92	0.34
Error	76	121.06	1.59		
Total	80	8082.16		
*C. glabrata*					
Temperature (A)	1	284.34	284.34	214.56	0.00 *^c^
pH type (B)	1	28.20	28.20	21.28	0.00 *^d^
A × B	1	1.68	1.68	1.27	0.26
Error	116	153.73	1.33		
Total	120	22,374.42			

^a^ 37 °C (11.26 mm) > 25 °C (8.64 mm), ^b^ Neutral (10.58 mm) > Acid (9.33 mm), ^c^ 37 °C (15.30 mm) > 25 °C (12.03 mm), ^d^ Neutral (14.18 mm) > Acid (13.15 mm), * *p* < 0.05.

**Table 7:** Comparison of the sizes of the growth-inhibition zones between the different concentrations of AMPH-B and types of moisturizers used against *C. albicans*.

		AMPH-B 1.25: 11.68 (0.30)	AMPH-B 0.63: 10.54 (0.15)	AMPH-B 0.31: 10.01 (0.09)	AMPH-B 0.16: 9.72 (0.32)	AMPH-B 0.08: 9.22 (0.15)	AMPH-B 0.04: 8.38 (0.16)	DMSO: 8.30 (0.17)
Liquid moisturizer	c-25 °C: 8.01 (0.11)	0.00 *	0.00 *	0.00 *	0.00 *	0.00 *	***0.22***	***0.79***
	c-37 °C: 10.45 (0.16)	0.00 *	***1.00***	***0.00 #***	***0.00 #***	***0.01 #***	***0.00 #***	***0.00 #***
	j-25 °C: 7.22 (0.11)	0.00 *	0.00 *	0.00 *	0.00 *	0.00 *	0.00 *	0.00 *
	j-37 °C: 9.98 (0.10)	0.00 *	0.00 *	***1.00***	***0.35***	***0.00 #***	***0.00 #***	***0.00 #***
Gel moisturizer	C-25 °C: 8.57 (0.12)	0.00 *	0.00 *	0.00 *	0.00 *	0.00 *	***0.90***	***0.35***
	C-37 °C: 11.16 (0.11)	0.00 *	***0.00 #***	***0.00 #***	***0.00 #***	***0.00 #***	***0.00 #***	***0.00 #***
	G-25 °C: 11.55 (0.12)	0.00 *	***0.00 #***	***0.00 #***	***0.00 #***	***0.00 #***	***0.00 #***	***0.00 #***
	G-37 °C: 15.00 (0.07)	***0.00 #***	***0.00 #***	***0.00 #***	***0.00 #***	***0.00 #***	***0.00 #***	***0.00 #***
	H-25 °C: 8.94 (0.12)	0.00 *	0.00 *	0.00 *	0.00 *	***0.28***	***0.00 #***	***0.00 #***
	H-37 °C: 11.39 (0.08)	***0.2***	***0.00 #***	***0.00 #***	***0.00 #***	***0.00 #***	***0.00 #***	***0.00 #***
	O-25 °C: 7.81 (0.08)	0.00 *	0.00 *	0.00 *	0.00 *	0.00 *	0.00 *	0.00 *
	O-37 °C: 10.58 (0.07)	0.00 *	***1.00***	***0.00 #***	***0.00 #***	***0.00 #***	***0.00 #***	***0.00 #***
	Q-25 °C: 7.76 (0.11)	0.00 *	0.00 *	0.00 *	0.00 *	0.00 *	0.00 *	0.00 *
	Q-37 °C: 10.68 (0.10)	0.00 *	***1.00***	***0.00 #***	***0.00 #***	***0.00 #***	***0.00 #***	***0.00 #***
	S-25 °C: 7.66 (0.08)	0.00 *	0.00 *	0.00 *	0.00 *	0.00 *	0.00 *	0.00 *
	S-37 °C: 10.85 (0.03)	0.00 *	***0.09***	***0.00 #***	***0.00 #***	***0.00 #***	***0.00 #***	***0.00 #***

The value in the box represents the *p*-value of the Tukey test, and in the horizontal direction, * represents that the size of the growth-inhibitory zone of AMPH-B was larger than that of the oral moisturizer, and # represents the opposite. The bold and italic values indicate that the size of the growth-inhibitory zones of the oral moisturizer is equal or greater than that of AMPH-B.

**Table 8:** Comparison of the sizes of the growth-inhibition zones between the different concentrations of AMPH-B and types of moisturizers used against *C. glabrata*.

		AMPH-B 1.25: 15.97 (0.06)	AMPH-B 0.63: 13.46 (0.03)	AMPH-B 0.31: 11.53 (0.02)	AMPH-B 0.16: 11.26 (0.02)	AMPH-B 0.08: 10.54 (0.05)	AMPH-B 0.04: 9.77 (0.06)	DMSO: 9.37 (0.07)
Liquid moisturizer	c-25 °C: 12.40 (0.07)	0.00 *	0.00 *	***0.00 #***	***0.00 #***	***0.00 #***	***0.00 #***	***0.00 #***
	c-37 °C: 15.77 (0.05)	***0.06***	***0.00 #***	***0.00 #***	***0.00 #***	***0.00 #***	***0.00 #***	***0.00 #***
	h-25 °C: 12.36 (0.03)	0.00 *	0.00 *	0.00 *	0.00 *	***0.00 #***	***0.00 #***	***0.00 #***
	h-37 °C: 15.89 (0.04)	***1.0***	***0.00 #***	***0.00 #***	***0.00 #***	***0.00 #***	***0.00 #***	***0.00 #***
	j-25 °C: 11.97 (0.06)	0.00 *	0.00 *	***0.00 #***	***0.00 #***	***0.00 #***	***0.00 #***	***0.00 #***
	j-37 °C: 14.84 (0.03)	0.00 *	***0.00 #***	***0.00 #***	***0.00 #***	***0.00 #***	***0.00 #***	***0.00 #***
Gel moisturizer	C-25 °C: 10.32 (0.04)	0.00 *	0.00 *	0.00 *	0.00 *	0.03 *	***0.00 #***	***0.00 #***
	C-37 °C: 14.57 (0.10)	0.00 *	***0.00 #***	***0.00 #***	***0.00 #***	***0.00 #***	***0.00 #***	***0.00 #***
	E-25 °C: 10.50 (0.07)	0.00 *	0.00 *	0.00 *	0.00 *	***1.0***	***0.00 #***	***0.00 #***
	E-37 °C: 14.57 (0.07)	0.00 *	***0.00 #***	***0.00 #***	***0.00 #***	***0.00 #***	***0.00 #***	***0.00 #***
	G-25 °C: 15.61 (0.14)	0.00 *	***0.00 #***	***0.00 #***	***0.00 #***	***0.00 #***	***0.00 #***	***0.00 #***
	G-37 °C: 18.58 (0.14)	***0.00 #***	***0.00 #***	***0.00 #***	***0.00 #***	***0.00 #***	***0.00 #***	***0.00 #***
	H-25 °C: 11.93 (0.07)	0.00 *	0.00 *	***0.00 #***	***0.00 #***	***0.00 #***	***0.00 #***	***0.00 #***
	H-37 °C: 14.93 (0.07)	0.00 *	***0.00 #***	***0.00 #***	***0.00 #***	***0.00 #***	***0.00 #***	***0.00 #***
	L-25 °C: 11.41 (0.04)	0.00 *	0.00 *	0.00 *	***0.61***	***0.00 #***	***0.00 #***	***0.00 #***
	L-37 °C: 14.41 (0.04)	0.00 *	***0.00 #***	***0.00 #***	***0.00 #***	***0.00 #***	***0.00 #***	***0.00 #***
	N-25 °C: 10.84 (0.06)	0.00 *	0.00 *	0.00 *	0.00 *	***0.00 #***	***0.00 #***	***0.00 #***
	N-37 °C: 14.33 (0.04)	0.00 *	***0.00 #***	***0.00 #***	***0.00 #***	***0.00 #***	***0.00 #***	***0.00 #***
	O-25 °C: 11.87 (0.03)	0.00 *	0.00 *	***0.00 #***	***0.00 #***	***0.00 #***	***0.00 #***	***0.00 #***
	O-37 °C: 14.52 (0.15)	0.00 *	***0.00 #***	***0.00 #***	***0.00 #***	***0.00 #***	***0.00 #***	***0.00 #***
	Q-25 °C: 11.86 (0.04)	0.00 *	0.00 *	***0.00 #***	***0.00 #***	***0.00 #***	***0.00 #***	***0.00 #***
	Q-37 °C: 14.86 (0.04)	0.00 *	***0.00 #***	***0.00 #***	***0.00 #***	***0.00 #***	***0.00 #***	***0.00 #***
	S-25 °C: 10.77 (0.16)	0.00 *	0.00 *	0.00 *	0.00 *	***0.01 #***	***0.00 #***	***0.00 #***
	S-37 °C: 14.76 (0.16)	0.00 *	***0.00 #***	***0.00 #***	***0.00 #***	***0.00 #***	***0.00 #***	***0.00 #***

The value in the box represents the *p*-value of the Tukey test, and in the horizontal direction, * represents that the size of growth-inhibitory zone of AMPH-B was larger than that of the oral moisturizer, and # represents the opposite. The bold and italic values indicate that the size of the growth-inhibitory zones of the oral moisturizer is equal or greater than that of AMPH-B.
